# Map2Check: Using Symbolic Execution and Fuzzing (Competition Contribution)

**DOI:** 10.1007/978-3-030-45237-7_29

**Published:** 2020-03-13

**Authors:** Herbert Rocha, Rafael Menezes, Lucas C. Cordeiro, Raimundo Barreto

**Affiliations:** 8 Johannes Kepler University, Linz, Austria; 9 University of Birmingham, Birmingham, UK; 10 Department of Computer Science, Federal University of Roraima, Boa Vista, Roraima Brazil; 11 Department of Computer Science, University of Manchester, Manchester, UK; 12 Institute of Computing, Federal University of Amazonas, Manaus, Amazonas Brazil

## Abstract

Map2Check is a software verification tool that combines fuzzing, symbolic execution, and inductive invariants. It automatically checks safety properties in C programs by adopting source code instrumentation to monitor data (e.g., memory pointers) from the program’s executions using LLVM compiler infrastructure. For SV-COMP 2020, we extended Map2Check to exploit an iterative deepening approach using LibFuzzer and Klee to check for safety properties. We also use Crab-LLVM to infer program invariants based on reachability analysis. Experimental results show that Map2Check can handle a wide variety of safety properties in several intricate verification tasks from SV-COMP 2020.

## 1 Overview

Fuzzing involves providing random data as input to a program and then checks for crashes. By contrast, path-based symbolic execution is an entirely static method that symbolically explores the program state-space [1]. Due to a focus on single runs, fuzzing techniques scale up relatively well. Path-based symbolic execution gives more confidence in the verification results, but it suffers from the path-explosion problem, thus limiting scalability. Here we exploit an iterative approach using fuzzing and symbolic execution to implement a tool named Map2Check v7.3.1 . Our main original contributions include: (i) use LibFuzzer [7] to provide random data as input to C programs to quickly expose "shallow" bugs, i.e., those that do not require complex data input; (ii) implement a new runtime library and instrumentation approach to monitor for crashes, failing built-in assertions and pointer safety; (iii) adopt Crab-LLVM [11] to infer invariants; (iv) exploit a sequential approach with LibFuzzer and KLEE [3] to check safety properties in a novel way; and (v) adopt MetaSMT as a wrapper around various SMT solvers, e.g., Boolector [2] and Yices [4], previously not supported by our tool. The SV-COMP'20 results show that Map2Check can be useful in both falsifying and proving reachability error and pointer safety-related properties.

⋆ Jury member

## 2 Verification Approach

Map2Check uses compiler techniques to analyze C programs using LLVM compiler infrastructure, thereby tracking pointer addresses and variable assignments in the LLVM bitcode [8]. In order to hold all values used in the analysis, a container API is employed in Map2Check. The tool also generates *built-in* assertions and checks them adopting an approach with fuzzing (to falsify properties) and symbolic execution (to prove the correctness). Fig. 1 illustrates the Map2Check flow, which has the following main steps: **(i)** convert the C code into the LLVM IR using Clang [5]; **(ii)** simplify the code via constant propagation and dead code elimination after the code instrumentation; (iii) to apply further Clang optimizations (e.g., canonicalize natural loops and promote memory to register); **(iii)** add Map2Check library functions to check the analyzed LLVM bitcode; **(iv)** generate inputs for Map2Check instrumented functions by executing LibFuzzer and then KLEE with Crab-LLVM; and **(v)** generate the witness file by identifying each basic block executed in the control-flow graph of the LLVM IR.

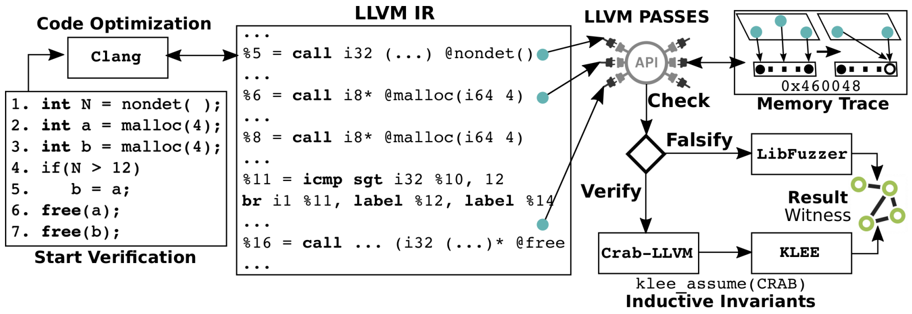

**Fig. 1.** Map2Check Verification Flow.

In order to explore the program states and to generate inputs for the Map2Check instrumented functions, the LibFuzzer implementation works by creating a custom entry point, which contains an array of bytes (of `uint8_t`). Thus, our implementation consists of generating concrete values from non-deterministic inputs that are our fuzzy targets. Additionally, we run multiple libFuzzer processes in parallel, where *N* fuzzing jobs should run to completion, i.e., until a bug is found or time/iteration limits are reached. Our fuzzing is coverage-guided (e.g., clang coverage), which tries to maximize the code coverage of a program. In our case, we adopted an `inline-8bit-counters` option from LLVM (SanitizerCoverage) for code coverage instrumentation built-in, where the compiler will insert inline counter that should be incremented on every edge.

The KLEE implementation works by creating a variable for the used data type, makes it symbolic, and then returns its value. As a result, KLEE produces concrete inputs for different program executions. We extend our KLEE implementation by adopting MetaSMT [6], which is an Embedded Domain Specific Language for SMT solvers. The API provided by MetaSMT is translated at compile-time, through template metaprogramming, into the native APIs provided by the SMT solvers [9]. Therefore, the overhead introduced by MetaSMT is small.

In order to improve the KLEE core solver execution, the KLEE tool is ran adopting: counterexample caching solver, which can be used to avoid calling the underlying solver in certain situations; and MetaSMT, which is employed to construct expressions that will be cached for each constraint to facilitate expression reuse. Note that symbolic execution often requires concrete solutions for satisfiable queries, e.g., before calling an external function, all symbolic bytes need to be replaced by concrete values, simplify constraints, and reuse query results [9]. Therefore, the KLEE cache solver is an important optimization, mainly of the counterexample cache that is based on the observation that many constraint sets are in a subset/superset relation.

To check the unreachability of an error location, we reduced the number of states in the analyzed program to be explored, thereby supplying invariants to the back-end solvers. We adopted Crab-LLVM [11] to infer inductive invariants as constraints to the error location. Therefore, the invariants are automatically introduced into the program as assumptions (before verification), and then KLEE receives the code as input. Crab-LLVM is a static analyzer that employs an abstract interpretation engine over LLVM bitcode based on the Crab library, which uses abstract domains such as intervals, octagon, and polyhedra. Crab is built on the top of IKOS[1] (Inference Kernel for Open Static Analyzers) to support a collection of abstract domains and fixpoint iterators.

## 3 Software Architecture

Map2Check v7.3.1 is implemented as a source-to-source transformation tool in C/C++ using LLVM (v6.0). Map2Check uses Clang (v6.0) as a front-end to parse a C program and to generate the respective LLVM bitcode to be used in the code transformation to track pointers and variable assignments. It uses LibFuzzer [7] (v6.0) and KLEE [3] (v2.0, as a symbolic execution) to automatically produce inputs to execute different program paths. MetaSMT (v4.*rc*2) is the API of reasoning engines. For SV-COMP'20, we adopt Yices (v2.5.1) that is used by KLEE to check constraints over bit-vectors and arrays, which substantially improved our results. Crab-LLVM [11] is used on reachability mode to infer inductive invariants for LLVM bitcode.

## 4 Strengths and Weaknesses of the Approach

Map2Check analyzed intricate verification tasks. The tool achieved the 2nd place in the ReachSafety-Arrays subcategory; in the ReachSafety-BitVectors category, Map2Check achieved a score of 46, thereby presenting better results than Pinaka, UKojak, VeriFuzz, and DIVINE. In other subcategories, our tool generated correct-unconfirmed and incorrect true results. These results are, in part, explained due to the Map2Check bugs in the witness generation and limitation to handle Crab-LLVM invariants from the over-approximations. We are investigating how to extend our tool by combining the data from fuzzing with KLEE as program assumptions using template invariant.

In the MemSafety category, Map2Check achieved a score of −68. However, our tool achieved essential results in comparison with the state-of-art tools, e.g., in the

[1] https://ti.arc.nasa.gov/opensource/ikos/

MemSafety-heap subcategory achieved a score of 174, which outperforms UAutomizer, ESBMC, DIVINE, and CBMC. Most incorrect results are, in part, explained due to bugs in the pointer tracking from our memory model, which could be improved by a trace semantics with program optimizations as relations on sets of the trace. Sadly, in the NoOverflows category, the score was −89. The incorrect results are, in part, explained due to bugs in the overflow analyzer. One way to improve this result is by combining the CPU flag postcondition test (LLVM supports several intrinsic functions, e.g., an add operation returns a structure with the result and overflow flag) with Sanitizers checking.

## 5 Tool Setup and Configuration

In order to run our `map2check-wrapper.py` script [10],[2] one must set the property file (`-p`) and the verification task; it provides as result: *TRUE* + *Witness, FALSE* + *Witness*, or *UNKNOWN*. For each error-path or correctness witness, a file (called `witness.graphml`) with the witness proof is generated in the Map2Check root-path folder. The dependencies, e.g., Clang and Yices tools, are included in the Map2Check distribution. The Benchexec tool info module is named `map2check.py` and Map2Check participates in SV-COMP'20 (as in the `map2check.xml` benchmark definition) in the following categories: ReachSafety-Arrays, ReachSafety-BitVectors, ReachSafety-ControlFlow, Reach Safety-Heap, ReachSafety-Loops, ReachSafety-Recursive, MemSafety, and NoOverflows.

## 6 Software Project

Map2Check v7.3.1 [3] is open source software distributed under the GPL license. We provide instructions for building Map2Check from the source in the file README (including the description of all dependencies). Map2Check is a joint project with the Federal University of Roraima and the Federal University of Amazonas in Brazil.
